# Tribological performance of high chromium white cast iron and heat-treated steel used in barite crushing industry

**DOI:** 10.1038/s41598-023-29627-4

**Published:** 2023-06-07

**Authors:** F. Zouch, A. Bahri, Z. Antar, K. Elleuch

**Affiliations:** Department of Materials Engineering, Laboratory of Materials Engineering and Environment (LGME), National Engineering School of Sfax, University of Sfax, B.P.W.1173, 3038 Sfax, Tunisia

**Keywords:** Engineering, Materials science

## Abstract

Barite sulfate (BaSO_4_) is considered a very important mineral material employed as a weighting agent for all types of drilling fluids. Meanwhile, crushers used for the grinding step during barite crushing are affected by catastrophic wear damage located in the hammer parts made from high chromium white cast iron (HCWCI). In the present study, a comparison of the tribological performance between HCWCI and heat-treated steel AISI P20 was conducted to investigate the possible substitution of HCWCI. The tribological test was performed under normal loads between 5 and 10 N for different durations (60, 120, 180, and 240 min). The wear response analysis for both materials showed that the friction coefficient increases as the applied load increases. Moreover, AISI P20 presented the lowest value compared to that attributed to HCWCI in all conditions. Furthermore, the analysis of the wear track obtained by means of scanning electron microscopy (SEM) revealed that the damage was an abrasive wear phenomenon for HCWCI with detection of a crack network throughout the carbide phase, which was more pronounced under the highest load. Regarding AISI P20, an abrasive wear mechanism was detected, characterized by several grooves and ploughing phenomena. Further, the analysis of the wear track using 2D profilometry revealed that for both loads, the maximum wear depth of the HCWCI wear track was significantly greater than that of AISI P20. As a result, when compared to HCWCI, AISI P20 exhibits the best wear resistance. Furthermore, as the load increases, the wear depth and the worn area increase as well. Also, the wear rate analysis supports the previous findings, which showed that under both loads, AISI P20 was more robust than HCWCI.

## Introduction

**Figure 1:**
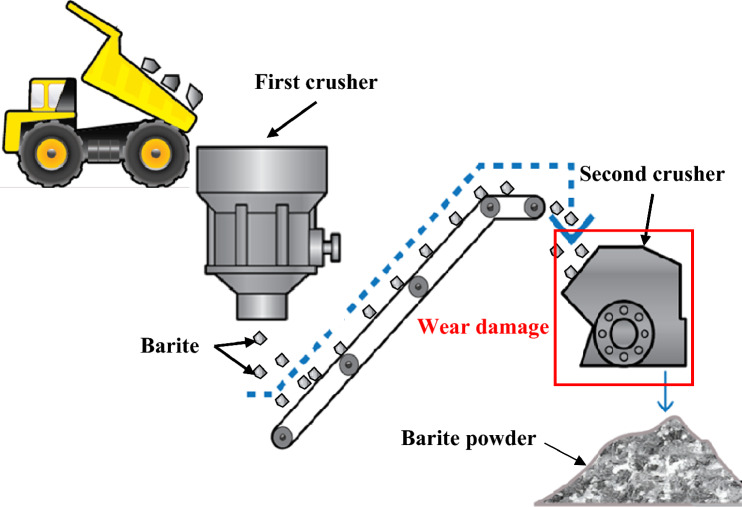
Barite-crushing process.

**Figure 2:**
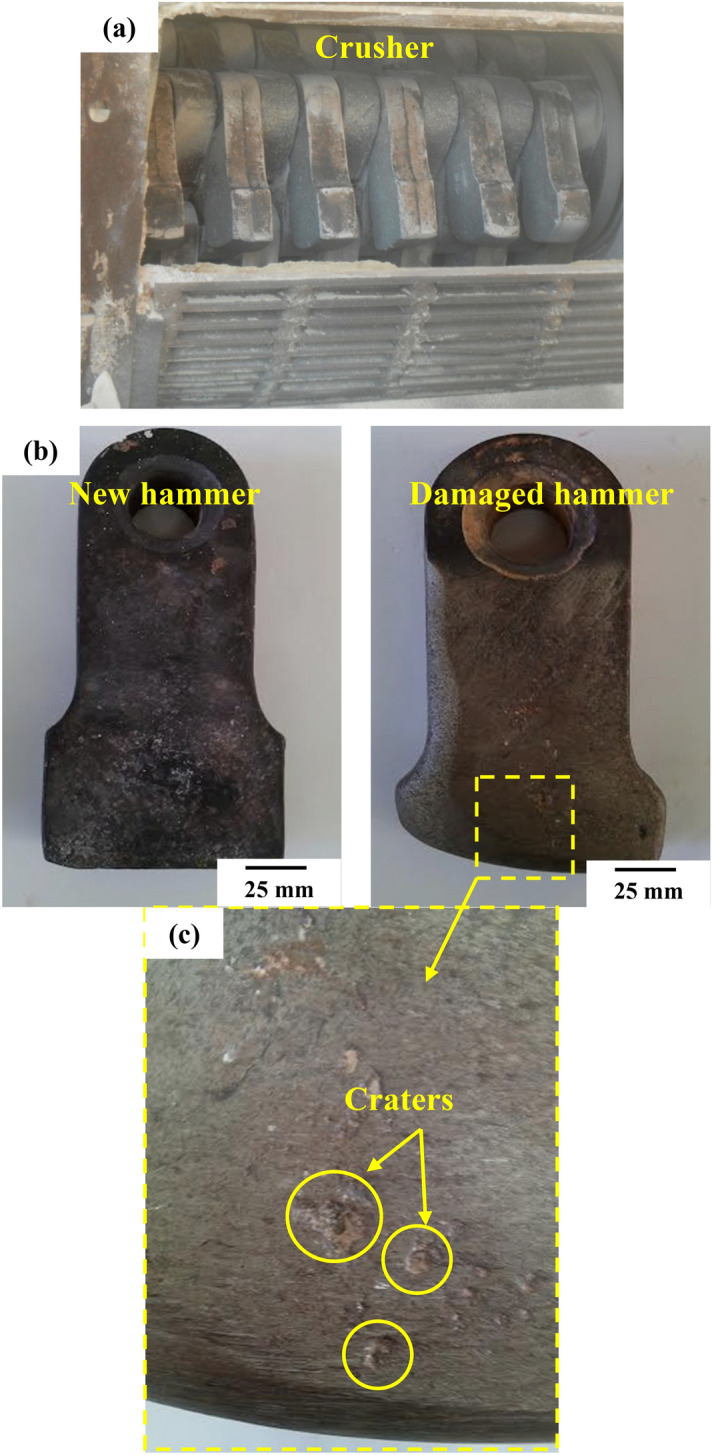
Crushing process: (**a**) Crusher, (**b**) Hammer before and after damage, and (**c**) wear damage.

The mineral product barite, also known as barium sulfate, has the chemical formula "BaSO_4_". The name of this substance refers to its tremendous density. The word "barite" actually derives from the Greek word "barys", which means "heavy", due to the element barium's high atomic weight, which is equal to 4.48 g/cm^3^ at a temperature of 26 °C. The fact that this product is widely consumed worldwide should not be overlooked. Indeed, the oil and gas sectors, which are the main industries for the usage of barite due to an uncommon combination of its features such as its high density, softness, and chemical inertness, are the principal beneficiaries of this global use. The other applications mainly focus on radiation protection and the chemical industry. It is important to mention here the steps required to produce barite powder, which are shown in Fig. 1. However, due to the high production rate and the challenging working conditions, various mechanical and tribological issues (Fig. 2) arise throughout this crushing process, ultimately causing process failure. These issues result in time and financial losses, a slowdown in manufacturing, and poor quality in the final product.

In the same context, several researchers investigated the damage of metallic materials^1–5^. In fact, Arabnejad et al. examined the effect of erodent particles hardness on the stainless steel and came to the conclusion that the erosion ratio rises as particle hardness increases^2^. Laguna-Camacho et al. analyzed the 304 stainless steel's erosion phenomena. The wear response demonstrates that the stainless steel 304’s erosion wear mechanism can be described by a brittle fracture of several big fragments with a 90-degree impact angle^4^.

However, literature studies dealing with the wear damage of the crusher in the industry are uncommon^6–10^. The latter^7,9^ focus their attention on the wear damage of the crusher used in olive oil extraction and barite industry. An overall investigation of the surface damage on the HCWCI employed in the crushers of barite rock reveals continuous, deep, and wide grooves that are separated by ridges brought on by the wear debris generated in the surface contact. Because of the continuous impact of barite particles on the hammer, numerous craters and a crack network are also observable on the damaged surface. As a result, it is possible to infer that the wear is a result of both abrasion and impact phenomena^9^.

Furthermore, Bahri et al.^7^ reported in their research on the extraction of olive oil that an abrasion and impact wear phenomenon occurred during the extraction process as a result of the repeated impacts of olive seed particles on the material surface. In fact, this damage is manifested by the presence of ploughing phenomena leading to the removal of material, pitting action, and also some grooves which are observed as a consequence of the detachment of large fragments.

Some researchers focused their work on the tribological study of HCWCI. In fact, Scandian et al.^11^ analyzed the relationship between the microstructure and the wear behavior of high chromium white cast iron using a pin-on-disc tribometer at room temperature under V = 0.31 m/s and F = 20 N. The analysis of the results reveals that the microstructure has a strong effect on the wear resistance of the material. Indeed, the wear loss is more important with a matrix that is fully ferritic than multiphase. Moreover, Fernández et al.^12^ investigated the wear behaviors of high chromium white cast iron with high and low carbon content. It was found that during the wear test, the damage of both materials started at the beginning with a plastic deformation phenomenon due to the compression of the contact area, which led to the apparition of several cracks. The latter leads to the fragmentation of the material into small particles. Also, Coronado^13^ analyzed the wear of white cast iron using abrasive alumina grains under various applied loads (from 2 to 15 N) at a constant speed of V = 66 rpm. The current study demonstrates that mass loss increases as the normal load increases. Further, it shows the presence of plastic deformation in the matrix, which is revealed by the SEM analysis of the worn surface of the longitudinal and transverse specimens.

It was also reported that some cracks are taking place in the carbide M_3_C form an angle of around 45 degrees with the scratches when the maximum load equal to 15 N is applied.

Other researchers investigated the wear behavior of AISI P20 steel^14,15^. Indeed, Lopes et al.^14^ studied the micro-abrasive wear of P20 steel. The analysis of the wear scar reveals the presence of an abrasive wear mechanism. Moreover, the wear mode is a combination of rolling and scratching phenomena. Oxidation wear was also presented due to the exposure of the wear debris generated during the wear test.

In the same context, Pereira et al.^15^ elaborated a comparative study between the wear resistance of AISI P20 steel after nitrogen treatment and after deposition of a cobalt alloy. Abrasive wear tests were conducted following ASTM G65-91 standards. The analysis of the result shows that the volume loss of the sample increases with the increase of both abrasive flow and the applied load. Moreover, it is important to note that the effect of the latter is more significant than that attributed to the abrasive flow.

In this respect, the AISI P20 steel in the current study underwent a particular heat treatment in order to improve its mechanical and tribological qualities. According to the literature, heat treatment actually improves the qualities of mild steel such as ductility, toughness, hardness, and tensile strength, as mentioned by Singh^16^.

Moreover, Chen et al.^17^ demonstrated that the mechanical characteristics of austenitic stainless steel 316 L were improved after heat treatment, which are mostly due to the number of phases that constitute this material as well as their morphologies.

The aim of this work is to perform a comparative study between HCWCI and AISI P20 in order to reduce the huge wear loss caused by the crushing process. To accomplish this task, a tribological study of those materials under different loads and test durations were conducted.

## Materials and methods

In the current study, 40 × 40 × 4 mm^3^ of both HCWCI and AISI P20 samples were prepared using a robot-wire machine for cutting. In fact, HCWCI were provided by SOFAP Company for further tests analysis. Regarding AISI P20, it was purchased from an industrial manufacturer.

It is worth noting that the chemical composition of HCWCI was determined using a spectroscopic metal analysis (Jobin Yvon JY 48®). However, the chemical composition of AISI P20 was determined by the technical data provided by the supplier. Table 1. illustrates the chemical composition of both materials.

**Table 1:** Chemical composition of HCWCI and AISI P20 steel.

HCWCI (wt%)								
C	Cr	Si	Mn	Mo	Ni	S	P	Fe
3.27	25.3	0.25	0.82	0.82	0.2	0.02	0.02	Ba

AISI P20 (wt%)								
C	Cr	Si	Mn	Mo	S	Fe		
0.40	1.90	0.40	1.50	0.20	0.08	Ba		

It is important to note that before the wear test, AISI P20 steel was undergone a heat treatment to enhance its mechanical and tribological properties. Indeed, the adopted heat treatment was performed according to the supplier's database. It is carried out at a temperature of 850 °C for 20 min, followed by oil quenching. Finally, a tempering step was realized to minimize residual stresses.

Prior to the wear test, each substrate's surface was well cleaned. To achieve a smooth surface with a low roughness equal to Ra = 0.06 m measured using 2D profilometry (Surtronic 25-Taylor Hobson), both HCWCI and heat-treated steel AISI P20 were mechanically polished with SiC papers. The surfaces were then degreased with an acetone solution to remove any impurities.

Moreover, a micro-hardness tester “Fisher Hardness Tester” was employed to measure the micro-hardness of both materials, which are equal to HV_0.05_ = 742 and HV_0.05_ = 702 attributed to HCWCI and AISI P20, respectively.

An optical microscope type (ZEISS-Axiotech) equipped with (ProgRes SpeedXT^core^ 5) camera was used to examine the microstructure of heat-treated steel AISI P20. However, HCWCI microstructure was identified by the use a scanning electron microscope (SEM, Jeol JSM-5410). To accomplish this task, both specimens were prepared by first being polished using SiC grinding papers, followed by an etching step of 10 s in Nital solution (3%) for heat-treated steel and 3 min in Nital solution (4%) for HCWCI.

HCWCI and heat-treated steel AISI P20 were underwent a tribological investigation using a rotating ball-on-disc tribometer. Based on the literature^18,19^, an alumina ball (Al_2_O_3_) was used as a counter-body. In our study, an alumina ball with a diameter of 10 mm was chosen.

The latter was selected as the counter-face material for a reason—its unique mechanical performance. According to published research^20,21^, alumina actually has a significant hardness that can reach more than 1400 HV and a surface roughness of Ra = 0.02 µm^22,23^. Furthermore, this material exhibits exceptional damage resistance^22^.

The wear tests were carried out using a ball-on-disc configuration under two normal loads of 5 N and 10 N at a velocity of V = 0.31 m/s for various test durations (t = 60, 120, 180, and 240 min). Therefore, all of the wear experiments were conducted under dry sliding conditions at ambient temperature (25 °C). After each wear test, substrates were cleaned with an acetone solution to eliminate the wear debris generated on the surface.

After the wear test, different characterizations were performed to determine the wear behavior of the specimens. In fact, the morphology as well as the chemical composition inside and outside the wear track were determined by scanning electron microscope SEM (FEI QUATRO) and energy-dispersive X-ray spectroscopy (EDX) techniques, respectively. Moreover, the 2D profilometer machine (Surtronic 25-Taylor Hobson) was used to quantify the wear.

Based on the literature^24^, the wear rate ($$K)$$ was calculated using the following Eq. (1):

1 $$K=\frac{V}{F\times D}$$

where F: Normal load, D: Sliding distance.

The materials loss volume of worn substance was determined using the Eq. (2):

2 $$V=2 \pi R S$$

where R is the radius of the wear track; S: the average of the cross-sectional area of the wear track. R and S were measured by a 2D profilometer machine. Three measurements of the cross-sectional area were conducted, and the results were averaged.

## Results and discussion

### Microstructure characteristics

**Figure 3:**
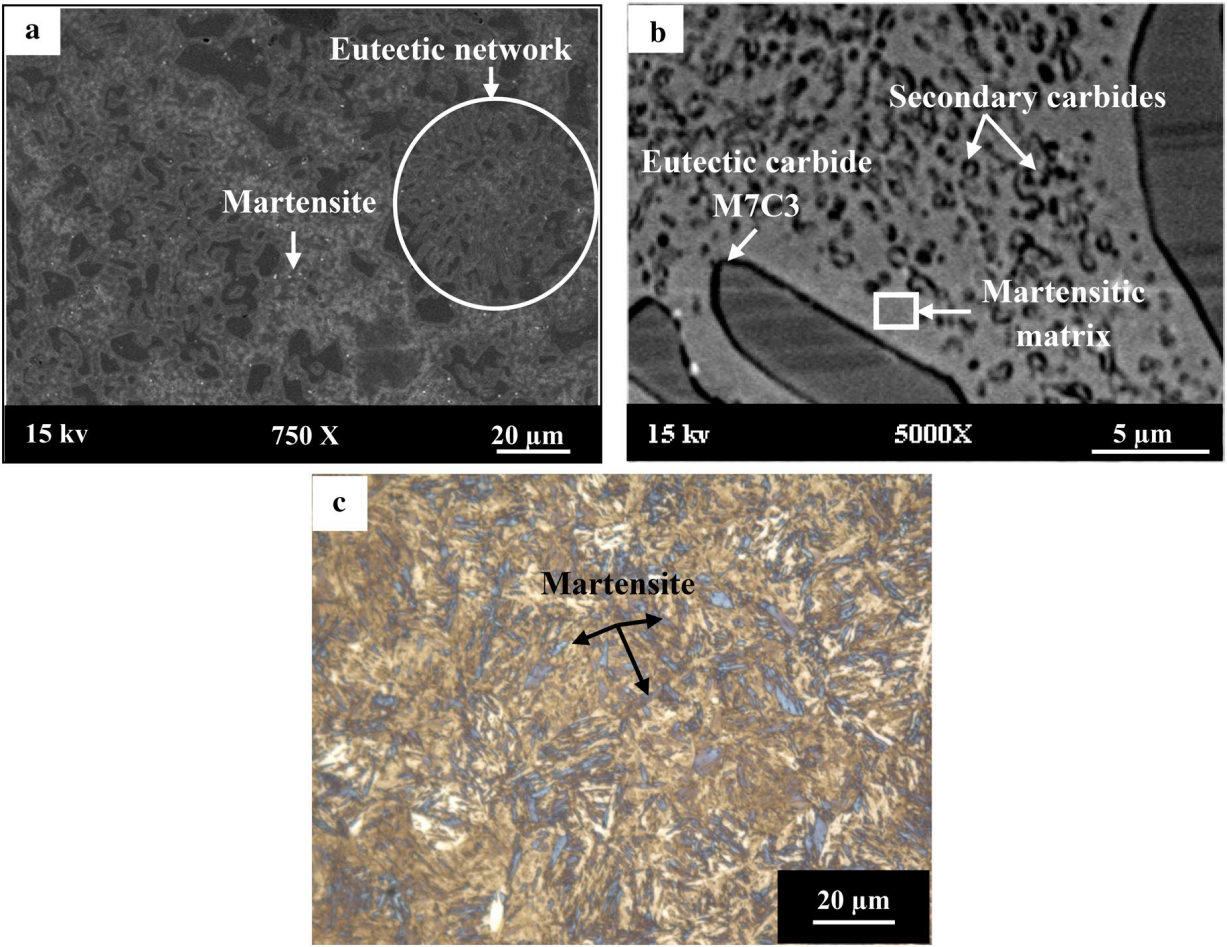
Microstructure of: (**a**) HCWCI by SEM, (**b**) magnification of (**a**) and (**c**) optical microscopy of heat-treated steel AISI P20.

Figure 3 presents the microstructure of HCWCI by using secondary electron imaging and heat-treated steel AISI P20 under optical microscopy. The analysis of Fig. 3a attributed to HCWCI, revealed the presence of a eutectic network within a martensitic matrix. Furthermore, the magnification in Fig. 3a and b demonstrates that the microstructure of the HWCI is composed of secondary carbides that have precipitated in a martensitic matrix. A similar microstructure was observed by Karantzalis et al.^25^ who studied the effect of heat treatments and alloying additions on the microstructure and properties of high chromium cast irons. However, a whole martensitic microstructure was detected upon analysis of the optical micrograph of heat-treated steel AISI P20 (Fig. 3c). Our findings are consistent with those of Priyadarshini et al.^26^. Indeed, they reported in their study that the microstructure of AISI P20 was characterized by a hard martensitic phase which was developed after direct quenching.

### Friction analysis

**Figure 4:**
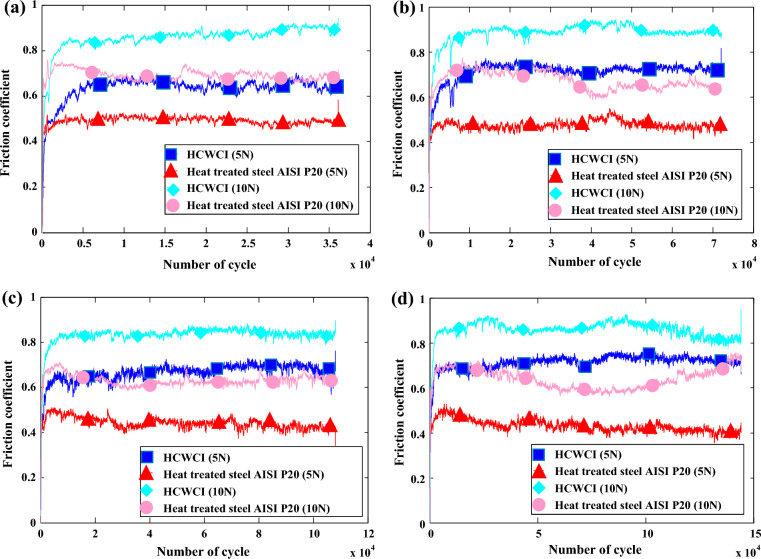
Friction coefficient evolution of both HCWCI and AISI P20 under: (**a**) t = 60 min, (**b**) t = 120 min, (**c**) t = 180 min and (**d**) t = 240 min.

The friction evolution of HCWCI and heat-treated steel AISI P20 involves two stages, according to the analysis of the friction coefficient presented in (Fig. 4). The tests were conducted with two normal loads equal to F = 5 N and 10 N at a velocity equal to V = 0.31 m/s in a dry condition. The friction coefficient rises significantly to a maximum value in the first, or "transition" stage. It remains constant for the entire specimen during the second stage, known as the steady-state, with slight oscillations that may be caused by debris formed in the wear track^18^. Furthermore, it is clear in Fig. 4 that increasing the load from 5 to 10 N raised the friction coefficient for HCWCI from 0.7 to 0.9 and for heat-treated steel AISI P20 from 0.5 to 0.7. As a consequence, the wear resistance of heat-treated AISI P20 could be more interesting than the HCWCI. It is also observed that the friction coefficient increases with increasing applied load for both materials. This elevation was approved by several researchers’ studies^27,28^. Meanwhile, no significant effect of the number of cycles on the friction coefficient evolution was detected. It is important to note that the correlation between the normal load or the contact pressure and the friction coefficient was taken into account in several research studies^29,30^. In the present study, the increase in the normal load results in an increase in the friction coefficient of the HCWCI and AISIP20 due to several reasons: (i) Such tribological behavior can be related to the creation of wear debris generated by the increased contact surface when the normal load increases. Wear debris act as abrasive particles and increases the wear track; (ii) an increase in normal load generates frictional heat at the contact surface; and mechanical properties, such as material strength, may increase as a result of bonding^29^. The reason behind the increase in normal load is to detect the effect of the high pressure on the tribological properties of both HCWCI and AISIP20. During the barite crushing process, several particles came into contact with the materials at different levels of contact pressure, which can affect the damage behavior of the materials.

### Wear examination

**Figure 5:**
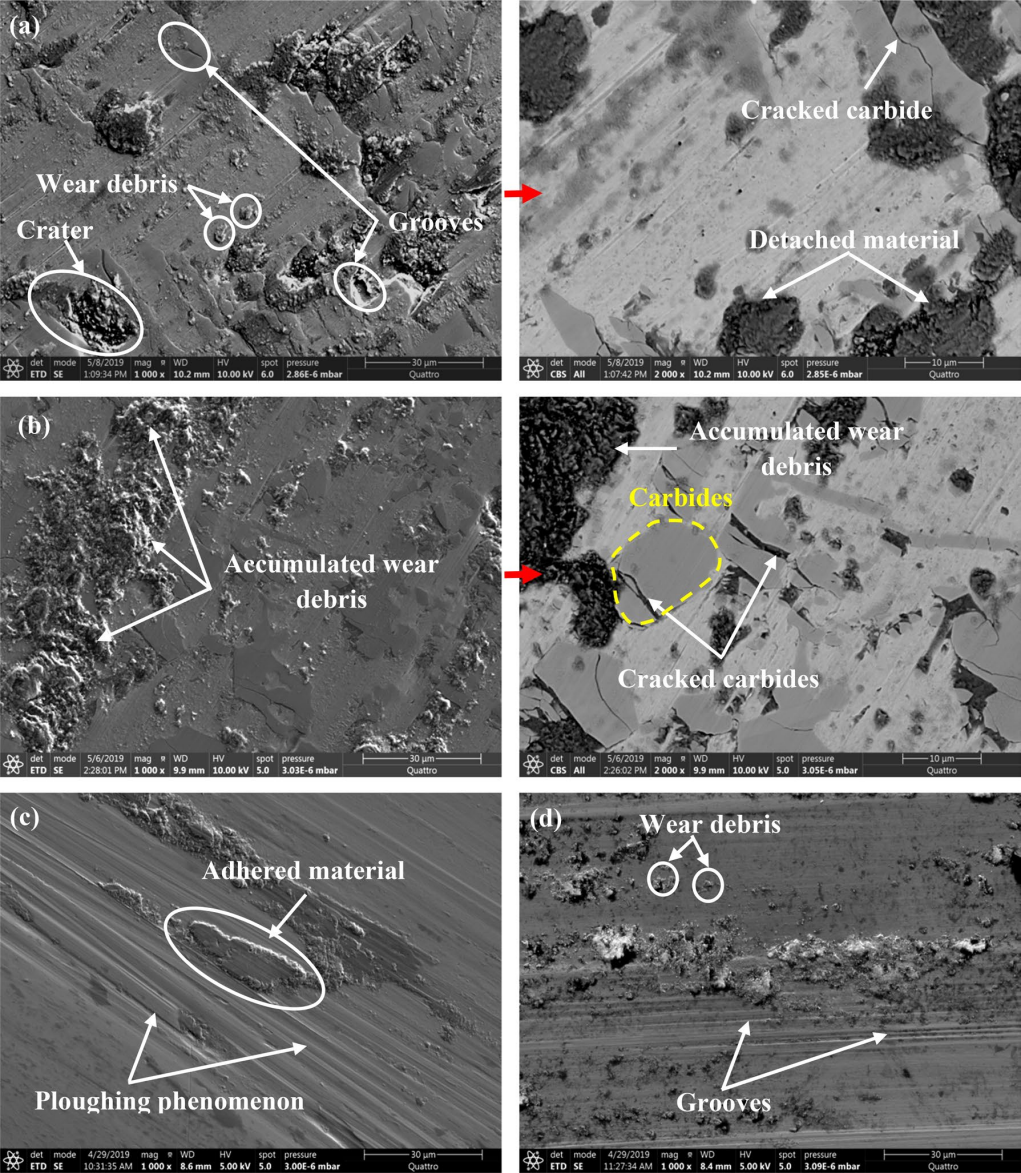
SEM micrograph of the wear track during t = 240 min (144,000 cycles). (**a**) HCWCI_F = 5 N, (**b**) HCWCI _F = 10 N, (**c**) heat-treated steel AISI P20_F = 5 N, and (**d**) heat-treated steel AISI P20_F = 10 N.

Figure 5 shows the SEM morphology of the wear track for both HCWCI and AISI P20. When analyzing the wear track of HCWCI, several wear phenomena can be detected. Figure 5a reveals the presence of wear debris located in the wear track, indicating that material detachment takes place during the tribological test under a normal load equal to 5 N. As a consequence, some grooves and craters can be observed. When increasing magnification, micro-cracks propagation was observed throughout the carbide phase; these micro-cracks increase material removal during friction. In previous research studies, similar results were obtained when analyzing the hammer wear damage during barite crushing^9^. It was reported that the intersection between radial and longitudinal micro-cracks throughout the carbide leads to the material removal, which previously undergo a plastic deformation^9,12^. These findings confirm that the tribological test reproduces the real wear damage of HCWCI. When applying a normal load of 10 N, the SEM micrograph (Fig. 5b) shows accumulated wear debris already detached from another area and plastically deformed during the wear test, leading to its accumulation under a high load. Furthermore, the SEM with high magnification was conducted, it is important to note that the increase in normal load leads to the creation of a crack network with deeper and wider micro-cracks. Regarding the wear track of AISI P20 presented in Fig. 5c, it was seen as a ploughing phenomenon, and the presence of grooves indicates an abrasive wear mechanism that is due to the creation of wear debris. The same wear phenomenon was found by Lopes et al.^14^ in their study about the micro-abrasive wear behavior study of carburization and ion plasma nitriding of P20 steel. Besides, the wear debris is adhered inside the wear track due to the plastic deformation during repetitive sliding. Similar aspects were found when applying a normal load equal to 10 N (Fig. 5d).

When comparing the obtained results for both HCWCI and AISI P20, it is worth noting that there is a difference in the wear aspects in terms of micro-cracks, craters, and ploughing phenomena. In terms of wear response, the effect of normal load is more pronounced for HCWCI than for AISI P20.

### Wear quantification

#### Profilometry analysis

**Figure 6:**
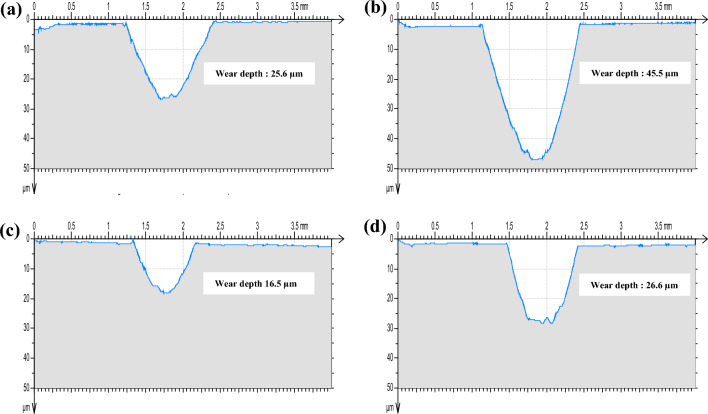
Wear track during t = 240 min (144,000 cycles). (**a**) HCWCI_F = 5 N, (**b**) HCWCI _F = 10 N, (**c**) Heat-treated steel AISI P20_F = 5 N, and (**d**) heat-treated steel AISI P20_F = 10 N.

Figure 6 presents 2D profiles of both materials, HCWCI and AISI P20 under normal loads equal to 5 N and 10 N.

It could be seen from the analysis of the obtained curves that the wear resistance attributed to AISI P20 is better than that related to HCWCI. The width and the maximum wear depth of the wear track of HCWCI are significantly more important than AISI P20 for both loads. Moreover, for each material, it is seen that the increase in load leads to an increase in the wear depth and the worn area of the wear track. As a consequence, it could be mentioned that the greater is the load, the greater is the material removal^31^.

#### Wear rate evolution

**Figure 7:**
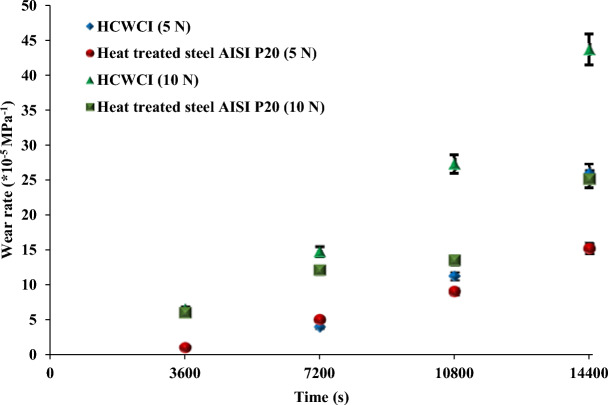
Wear rate evolution.

Figure 7 illustrates the variation of the wear rate of HCWCI and AISI P20 as a function of the test duration. It is clearly shown that all curves show similar trends. Indeed, the obtained results show that the wear rate increases with increasing test duration and normal load for both materials (Fig. 7). These findings are in good agreement with several research studies^32,33^. Hani Aziz et al.^32^ who studied the effect of the load on the wear rate of steel, aluminum, and brass, they mentioned that the increase in the load led to an increase of in wear rate for all materials. Moreover, Lakshminarayana et al.^33^ focused their research on the study of the effect of the load on the wear rate and the frictional resistance of EN-8 steel sliding against EN-31 steel. They found that the augmentation of the load from 20 to 200 N increased the wear rate from approximately 4 × 10^–4^ to 77 × 10^–4^ mm^3^/N m. For more explanation, the increase in the wear rate is the consequence of more material removal due to the elevation of the temperature in the contact area leading to a modification of the sample’s behavior to a ductile one by the increase in friction with the applied load.

Besides, it is clearly shown that the best wear resistance was retained for AISI P20 under both loads F = 5 N and F = 10 N due to the significant decrease of the wear rate between both of them.

**Figure 8:**
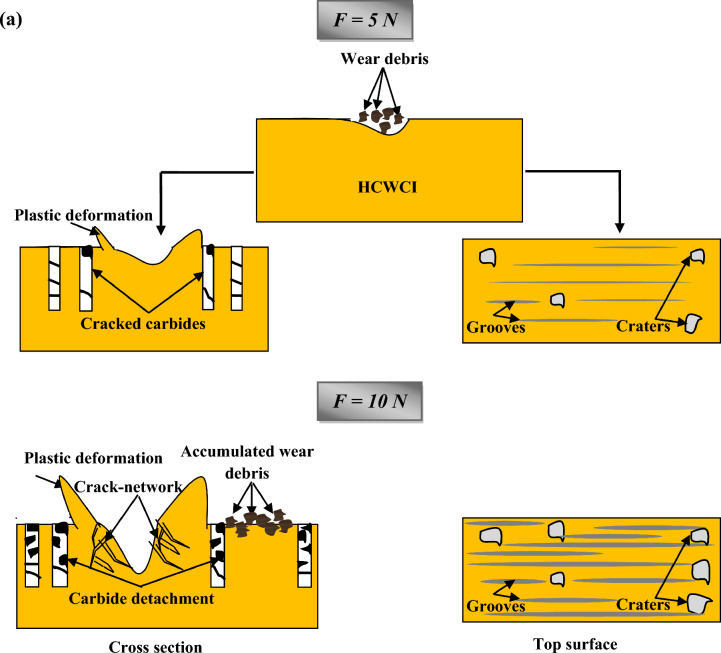

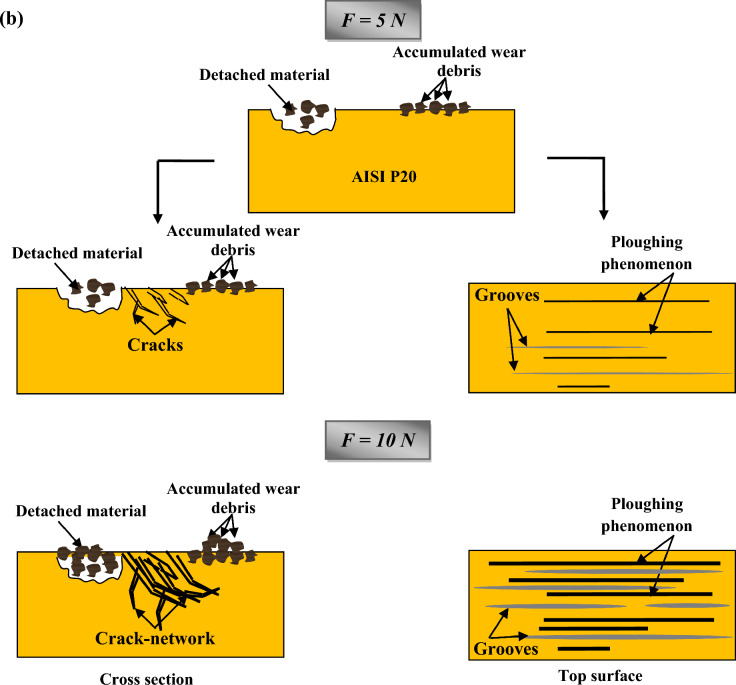
Wear mechanism evolution when increasing normal load from 5 to 10 N of: (**a**) HCWCI and (**b**) AISI P20.

Figure 8a and b illustrates a synthetic presentation of the wear mechanism evolution of HCWCI and AISI P20 when increasing the normal load from 5 to 10 N.

## Conclusion

In the present work, a comparative study between the tribological properties of both HCWCI and heat-treated steel AISI P20 has been conducted under dry conditions at ambient temperature (25 °C). The effects of applied load and test duration were experimentally studied. The following conclusions can be drawn according to the work conducted:

- The pin-on-disc test results under two loads F = 5 N and F = 10 N illustrate that the friction evolution of HCWCI and heat-treated steel AISI P20 consists of two stages. The first, or “transition” stage, the COF rapidly increases to reach a maximum value. During the second stage, or steady state, the friction keeps the same value for the entire specimen with the presence of some fluctuation, which could be due to the presence of some debris generated in the wear track. The analysis of the friction curves reports that HCWCI is characterized by the highest friction coefficient when compared with AISI P20 for all conditions.
- Increasing the load from F = 5 N to F = 10 N led to an increase in the COF values from 0.7 to 0.9 for HCWCI and from 0.5 to 0.7 for heat-treated steel AISI P20.
- The SEM morphology of the wear track of HCWCI shows different features of wear causing material loss. Indeed, micro-cracking, abrasive wear, and material detachment provoke severe damage.
- Regarding the wear track of heat-treated steel AISI P20, it was found that the damage combines both ploughing phenomena and the presence of grooves, indicating an abrasive wear mechanism that is due to the creation of wear debris.
- The analysis of the wear response mentioned that the effect of the normal load is more pronounced for HCWCI than attributing to AISI P20.
- The profilometry analysis indicates that the wear depth attributed to HCWCI is more important than heat-treated steel AISI P20 indicating that the latter presents the best wear resistance.
- Increasing the load from F = 5 N to F = 10 N increase the wear depth and the worn area, causing more damage.
- The wear rate increase with the increase of the applied normal loads.
- HCWCI presents the most important wear rate indicating that heat-treated steel AISI P20 has the best wear resistance.

Finally, HCWCI could be replaced with AISI P20 to improve wear resistance and reduce damage.

## Data Availability

The datasets used and/or analysed during the current study available from the corresponding author on reasonable request.
